# Bodily synchronization underlying joke telling

**DOI:** 10.3389/fnhum.2014.00633

**Published:** 2014-08-15

**Authors:** R. C. Schmidt, Lin Nie, Alison Franco, Michael J. Richardson

**Affiliations:** ^1^Department of Psychology, College of the Holy CrossWorcester, MA, USA; ^2^Center for the Ecological Study of Perception and Action, University of ConnecticutStorrs, CT, USA; ^3^Center for Cognition, Action and Perception, Department of Psychology, University of CincinnatiCincinnati, OH, USA

**Keywords:** motor movements, sensorimotor synchronization, social interaction, spectral decomposition

## Abstract

Advances in video and time series analysis have greatly enhanced our ability to study the bodily synchronization that occurs in natural interactions. Past research has demonstrated that the behavioral synchronization involved in social interactions is similar to dynamical synchronization found generically in nature. The present study investigated how the bodily synchronization in a joke telling task is spread across different nested temporal scales. Pairs of participants enacted knock–knock jokes and times series of their bodily activity were recorded. Coherence and relative phase analyses were used to evaluate the synchronization of bodily rhythms for the whole trial as well as at the subsidiary time scales of the whole joke, the setup of the punch line, the two-person exchange and the utterance. The analyses revealed greater than chance entrainment of the joke teller’s and joke responder’s movements at all time scales and that the relative phasing of the teller’s movements led those of the responder at the longer time scales. Moreover, this entrainment was greater when visual information about the partner’s movements was present but was decreased particularly at the shorter time scales when explicit gesturing in telling the joke was performed. In short, the results demonstrate that a complex interpersonal bodily “dance” occurs during structured conversation interactions and that this “dance” is constructed from a set of rhythms associated with the nested behavioral structure of the interaction.

## INTRODUCTION

When people play music and dance together, they visibly engage in shared rhythmic timing of their bodies (Keller and Rieger, 2009). Such rhythmic bodily coordination has also been noted in dyadic sports contests (e.g., McGarry et al., 2002; Palut and Zanone, 2005) even where competition is paramount. However, such interpersonal entrainment is not limited to explicit coordination tasks such as dance and sports. Researchers for some time have known that people also exhibit a bodily “dance” or a “correlated behavioral waves” (Newtson, 1993) in natural social interactions (Condon and Ogston, 1967; Davis, 1982). With the proper methodologies, the coupled activity waves are detectable for most common daily activity such as two people walking together, interacting as customer and cashier at a store or having a conversation (Schmidt et al., 2012, 2013; Dale et al., 2013).

Many cognitive researchers have turned to two-person neuroscience (Hari et al., 2013) to understand such joint actions and have approached social entrainment in particular by studying the brain-to-brain couplings and arguing that understanding such neuroentrainment processes will allow us to understand not only behavioral entrainment but how we generally “create and share a social world” (Hasson et al., 2012). Although there are methodological difficulties associated with imaging the activity of two brains simultaneously while people are interacting in that movement is typically constrained during fMRI and EEG recordings (Konvalinka and Roepstorff, 2012), new techniques and tasks afford promise. For example, Yun et al. (2012) used hyperscanning EEG to investigate implicit bodily coordination and assess the underlying neural correlates and the interpersonal functional connectivity between brain regions. They found that a training period in which one participant follows another’s random index finger movements led to post-training unconscious synchronization when the participants viewed each other’s “stationary” fingers—in fact, the participants could not keep their fingers stationary and their movements were unconsciously moving in a coordinated fashion. Moreover, they found that the overall number of significant inter-brain phase synchrony connections increased after training mainly in the inferior frontal gyrus, anterior cingulate, and parahippocampal gyrus which previously have been found related to processing of social information including theory of mind and social contextual cues (Amodio and Frith, 2006).

However, neural structures are always causally implicated in the exercise of all human capacities; and consequently, it is important to note that it is still unclear what causal role these neural processes play in activities such as social entrainment. They are, of course, a necessary condition for the behavior but not sufficient for understanding how *the whole person* acting. In order to fully understand a behavior, one needs to know not only the underlying neural processes but also the embodied situation within which a person is acting because behavior and mental states emerge from manifold interactions between the brain and the behavioral consequences on the environment. There has always been a tendency in behavioral theory to consider the neural level of constraint a proprietary level of explanation rather than conceive of the behavioral system as consisting of an embodied brain embedded in an environment where the behavior of *the whole person* emerges from the self-organized interaction across multiple space-time scales. Neural explanations risk committing a mereological fallacy by ascribing the activity of the whole person as a unity to the workings of an inner entity, namely the brain.

An alternative to the traditional neurocognitive explanation of behavior in general and social synchrony in particular comes from the behavioral dynamics perspective (Kelso, 1995; van Gelder, 1998; Smith and Thelen, 2003; Warren, 2006) whose goal is to identify general laws of pattern formation that govern the causal unfolding of human behavior rather than searching for neurophysiological loci of behavior generation. The behavioral dynamics perspective maintains that physical systems at any scale (chemical, neural, behavioral, social) can be understood in terms of how its components *balance* to form stable patterns, which can be characterized as equilibriums, steady-states of change or more generically as attractor states (Kugler et al., 1980). As such, the behavioral dynamics perspective suggests that dynamical similitude is a key property of natural systems generally: the dynamical organizational principles (such as synchronization of rhythms) are replicated at the different scales of nature and similar patterns should appear although the properties being organized by these principles will be scale dependent. Consequently, behavioral dynamics is generic enough to explain emergent patterns at different scales as well as the micro-to-macro mapping across those scales. Consequently, the neural level is not viewed then as a proprietary level of explanation of behavior but rather one necessary level of constraint, which interacts with processes of body, the physical environment as well as the social cultural environment, all of which make up an embodied and embedded behavioral system.

### BEHAVIORAL DYNAMICS PERSPECTIVE ON SOCIAL ENTRAINMENT

For more than two decades, human movement science researchers have approached social entrainment from a behavioral dynamics perspective (Schmidt et al., 1990, 2011; Oullier et al., 2008; Schmidt and Richardson, 2008). Research from this perspective uses concepts and tools from nonlinear dynamical systems (e.g., Strogatz, 1994; Kelso, 1995) to tackle social entrainment as an instance of self-organization, where individuals form a social unit, a dynamical interpersonal synergy (Marsh et al., 2006), without planning, to construct meaningful actions together. This means that behavioral entrainment in social interactions can be understood using same self-organizing processes used to understand the entrainment of mechanical oscillators (e.g., pendulum clocks, Huygens et al., 1673/1986). In this way, social coordination is cast as an instance of synchronization phenomena found generically in nature, and thereby, it can be measured (Oullier et al., 2008) and understood using mathematical models of synchronization (Pikovsky et al., 2001; Strogatz, 2003).

A number of studies (Schmidt and Turvey, 1994; Schmidt et al., 1998; Richardson et al., 2007) have found that if pairs of participants *intentionally* coordinate their arms or legs like people do in certain dance-like activities and that the patterns of coordination observed are indicative of an interpersonal synergy created by dynamical processes of synchronization (Haken et al., 1985; Kelso et al., 1990). Other studies (e.g., Schmidt and O’Brien, 1997; Neda et al., 2000; Richardson et al., 2005, 2007; Issartel et al., 2007; van Ulzen et al., 2008; Miles et al., 2010) investigated whether interpersonal dynamical synchronization would occur *spontaneously* without conscious awareness of the participants. For these studies, pairs of participants would perform incidental rhythmic movements (e.g., swinging a pendulum, clapping, walking) often while simultaneously performing some focal task (e.g., discriminating together the differences in the features of two similar cartoon faces). Results demonstrated that the incidental rhythmic movements spontaneously became synchronized and had greater phase entrainment than would be expected by chance. These unconscious synchronization studies speak to the generality of the dynamical processes in social coordination and provide support for the hypothesis that such dynamical processes underlie interpersonal synergies in everyday interactions.

By recording simple rhythmic movements, these laboratory studies were able to easily use standard time series analyses to evaluate the dynamical processes of synchronization involved in interpersonal coordination. However, the interactions involved were not very natural (i.e., swinging handheld pendulums). Consequently, these studies still leave unanswered the question of whether the dynamical movement synchrony occurs in natural, everyday social interactions (like conversations) where the bodily movements made are subtler and less overtly rhythmic.

Recent developments in psycholinguistics of dialog, however, suggest that the bodily movements seen in conversation emerge from an interpersonal synergy (Fowler et al., 2008; Fusaroli and Tylen, 2012; Dale et al., 2013; Fusaroli et al., 2013). Although this synergistic dialog work emphasizes linguistic coordination, the idea is that speakers in conversation synchronize non-linguistic actions such as prosody, manual gestures, eye gaze, posture, body movements, and deictic words in functional, dynamical, real-time sense. Schmidt et al. (2012) combined time series analysis and traditional social psychological measures of social entrainment to study the coordination of whole body movements or activity in a structured conversation task, namely, telling knock–knock jokes. Video recording of the interactions were analyzed using two methodologies, a rater-coding method and a computational video image method, to provide time series of the movements of the participants as they enacted a series of jokes. Analyses revealed spectral peaks indicative of nested periodicities at the time scale of the joke and near the time scale of the utterance. That is, there were activity rhythms associated with the whole joke (one occurring about every 8 s) and with the lines of the joke (one occurring about every 1–2 s). Moreover, not only were the activity waves in the time series significantly more correlated than expected by chance, but they also exhibited dynamical “inphase” synchronization demonstrating a tendency for teller and responder of the jokes to be active at the same time in spite of the turn-taking nature of the task. The results of Schmidt et al. (2012) indicate that the dynamical entrainment processes that have been used to model the interpersonal synchronization in more laboratory tasks seem to also be present in more everyday conversation tasks. Moreover, the results suggest that actors may have used the dynamical connection between their rhythms to predict or anticipate each other’s actions, and hence, may facilitate (or even obviate the need for) mental simulation processes proposed for this function (Sebanz and Knoblich, 2009).

### CURRENT STUDY

**FIGURE 1:**
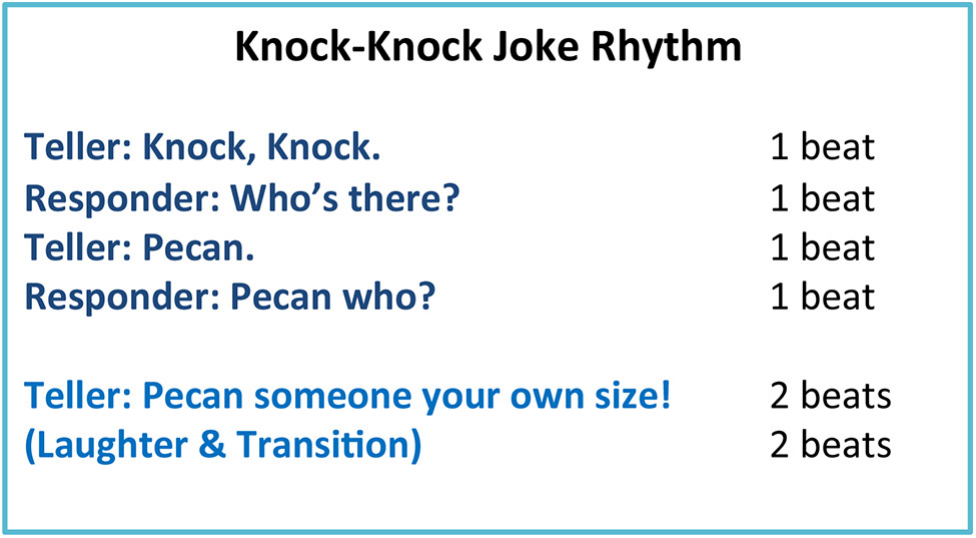
**Knock–knock jokes have an inherent rhythmic structure in how they are performed**.

The current study is a further investigation of bodily synchronization in the structured conversation task of joke telling used by Schmidt et al. (2012). One of the goals of the study is to further investigate the complexity of the “dance” underlying the activity rhythms of the joke teller and the responder. As noted above, periodicities were observed a multiple time scales in their activity time series. This is due to the nested temporal nature of the joke telling task (**Figure 1**). Each of the first four lines is said isochronously, 1 beat each for each line, comprising 4 beats for the setup of the joke; and typically the last (punch) line is given 4 beats (2 beats to say the line and 2 beats pause before the next joke) for the conclusion of the joke. Moreover, in Schmidt et al. (2012), four jokes were enacted in a row. Consequently, the joke sequence is comprised of a nested set of temporal events each of which has its own behavioral goal. The joke itself is the overall event/goal but it contains the set-up of the joke and punch line of the joke which are subgoals. The setup and the punch line themselves contain two-person exchanges in which each person says a line. However, two-person exchange event/goal contains the sub-goals of each person uttering one line of the joke. Rhythmically, the overall joke is comprised of 8 beats, the set-up and punch line are comprised of 4 beats each, a two-person exchanges takes two beats and each line (utterance) is one beat. The question is how the entrainment between teller and responder is spread across the time scales corresponding to each of these events. Are there subsidiary rhythms associated with each of these events that are interpersonally synchronized? In this study, we used a spectral decomposition technique called a wavelet analysis to evaluate this question to better appreciate the nested nature of the joke telling “dance.”

Investigating the temporal relatedness of people interacting at different time scales extends how we think about the relatedness of systems in time. Traditionally, we have looked at time as being past, present, and future and have been interested in how the past and future (for intentional systems) influence the present state of the system. Hence traditionally, the temporal relatedness of interacting components corresponds to whether they are correlated moment-to-moment at the present time or whether there is some time-lagged influence (e.g., past event effecting present moment). An alternative is to look at time as being made up of the hierarchically nested events of the physical process being observed—the “time as conflict” perspective (Fraser, 1978). From this perspective, the question of the relatedness of the interacting components is not only whether they are related in the present moment-to-moment but also whether and how they are related in terms of the hierarchically nested events that comprise the interaction at different time scales. Such a question of whether system components are related at different time scales associated with subgoals/events is not only important for this joke telling interaction but also any action or interaction since any action or interaction is typically performed within subgoals/events that unfold at a longer time scales. In the current joke telling task, the subgoals/events are perhaps more obvious because they are metrically related, and hence, easily demarcated. However, any action performed is typically nested within subgoals/events that unfold at a longer time scale. For example, swinging at a baseball is nested within a “time at bat” event, which is nested within an inning event, which is nested within a game event. Understanding this nested structure of subgoals/events, one can ask about how the dynamics unfold at each of these time scales and how system components (e.g., teams) are related (i.e., who is winning) at each of these time scales and also how the events unfolding at one time scale affect the events unfolding at a longer time scale [e.g., how the response to a given pitch is affected by previous pitches at bat; see Gray (2002)]. The importance for the present investigation of nested structure of subgoals/events in the joke task is whether interacting individuals are connected at these different and presumably psychologically real time scales and whether interactional “synergy” exists not just moment-to-moment but rather in the dynamic unfolding of both longer- and shorter-term events.

A second goal of the study was to understand the complementary roles played by the teller and responder in joke telling bodily entrainment. In Schmidt et al. (2012), the two participants exchanged roles every joke making it difficult to evaluate whether the difference in roles led to an asymmetry in the coordination. In the current study, one person remained the teller throughout the sequence of jokes. Interestingly, Yun et al.’s (2012) implicit interpersonal coordination study found an asymmetry in the interpersonal functional connectivity between brain regions such that the person whose movements had “followed” the leader’s movements in the training session had additional activation of areas related to socio-motor and motor-visual interactions. In the current study, we evaluate whether there was a leader–follower relationship apparent in the bodily activity of the teller and responder and whether this varied across the nested time scales of the joke.

A third goal of the study was to investigate the importance of visual information in creating interpersonal entrainment. Shockley et al. (2003) found that just verbal information (i.e., talking but not seeing) is sufficient for creating spontaneously coordinated postural sway of two conversing participants. To investigate whether this was the case during the structured joke telling task employed here, we had participants tell jokes while facing each other as well as facing away from each other. Of interest was whether adding visual information creates stronger synchronization between the teller and the responder and how this varied across the nested time scales of the joke.

A fourth goal of the study was to investigate how the magnitude of the participants’ movements’ influence the degree of behavioral entrainment in this structured conversation task. Schmidt et al. (2012) found that some of the participant pairs moved very little in enacting the jokes while others moved more. The question was whether gesturing more during an interaction necessarily led to more behavioral synchrony and how this varied across the nested time scales of the joke. To evaluate this, we instructed some of our participant pairs to gesture stereotypically while enacting the jokes.

A final goal of the study was to evaluate whether the degree of behavioral entrainment observed in a dyad was associated with the degree of the subjects’ social competence. Bodily entrainment dynamics identified in these dyadic interactions may be a fine-grained behavioral characterization of individuals’ awareness of each other and social conventions. Consequently, in addition to identifying the strength and patterns of the dyads’ entrainment dynamics, we also explored whether the degree of entrainment observed was related to self-report measures of social skills, namely, the Self-Monitoring scale and Autism Spectrum Quotient, that indexed a participant’s social competence.

## MATERIALS AND METHODS

### PARTICIPANTS

Thirty-two undergraduate students from the College of the Holy Cross ranging in age from 17 to 22 years participated in this study for partial course credit. The participants were combined into 16 pairs. Eight of the pairs were female pairs and the others were mixed-gender pairs. Eight pairs of the participants had never seen each other prior to the experiment; three pairs had seen each other on campus; two pairs knew each other well enough to speak in passing and only three pairs were friends prior to the experiment. The experiment was approved by the College of the Holy Cross Institutional Review Board. All participants provided informed consent.

### MATERIALS AND PROCEDURE

Upon arrival, the participants were told that the experiment was investigating the cognitive processes underlying leisure activities while the true aim of the study was to evaluate the process of their movement synchrony during joke telling. First, the participants were told to perform two standard team-work tasks. The tasks were intended to function as “ice breakers” so that the participants not only became comfortable with each other’s presence (Brue, 1985) but also became accustomed to coordinating their movements. Because past research has suggested that physical touch enhances cooperative behavior [see Kraus et al. (2010)], the tasks we chose required the participants in a pair to be in physical contact with each other. In a “stand-up” task, two participants began sitting on the ground, back-to-back, knees bent and elbows locked, and tried to stand up without falling down. In a “balloon” task, participants tried to move as many large-sized balloons as possible in one minute by carrying the balloons in between their bodies without using arms or hands to cross the laboratory space. The latter task was skipped for mixed-gendered pairs.

Next participant pairs enacted a series of ten knock–knock jokes. Schmidt et al. (2012) used only four jokes. The number was increased to ten in this study to increase the length of the interaction. The interactions were recorded using a Kinect for Windows camera (Microsoft Corporation, Redmond, WA, USA). The Kinect records video up to 30 frames per second and renders frame-to-frame the whole-body interactions of the participant pairs using skeletal frame models that specify the 3D locations of 21 body joints for each person. The Kinect camera was placed on a tripod at trunk-level approximately 2 m in front of the participants and exactly at the midline bisecting the distance between the two participants. Participants were led to their designated standing spots so that their positions were symmetrical of each other. The participants stood approximately 1 m apart. A 3 m wide, sliding, beige-colored curtain hung from the ceiling of the laboratory behind the participants to provide a uniform background for filming.

The person standing on the left side of the video image was always designated as the joke teller who initiated the knock–knock joke. The person on the right side of the video image was always the responder. Both participants remained in their roles throughout the recitation of the series of ten jokes. The lines of the jokes for the teller and the responder (**Figure 1**) were written, respectively, on two cards. The participants were asked to take a few moments to familiarize themselves with the joke’s lines and their concomitant puns in order to be comfortable telling the jokes. Participants could refer to the cards throughout the joke telling and did not need to memorize them.

The jokes were told while varying whether the participants had visual information about each other (facing toward or away) as well as to the extent that they explicitly gestured while telling the jokes (gesture or non-gesture). Each pair of participants enacted the jokes both facing toward each other first and facing away from each other. The order of facing direction was counterbalanced. In the facing away condition, the participants faced away at an angle of approximately 120^∘^ just enough that they could not see each other but so more of their body was in view of the camera. For both conditions, they were facing towards the camera so that a near-sagittal view of their bodies was in the camera frame to capture all their body movements. In addition, half of the participant pairs were told to explicitly use gestures with their hands, arms, and bodies as suited for the joke telling. For example, when the teller said “Knock—knock,” they were instructed to imitate the motion of knocking on a door. The listener, who responded “Who’s there?,” was instructed to shrug their shoulders and turn up the palms of their hands in a motion indicative of questioning. The experimenter demonstrated these specific gestures and encouraged the use of other gestures that aligned with the content of the jokes. The non-gesture group was not given any instructions on how to move but was told to recite the joke as they naturally would. Both groups of participants were, however, told they were welcome to laugh freely and express their emotions as they told the jokes. In addition to demonstrating the gesture or non-gesture versions of telling the jokes, the experimenters also demonstrated the pace of telling so that each joke takes approximately 5–7 s. To ensure natural postures and mobility, participants had their shoes removed throughout the experiment.

When participants verbally indicated that they were ready, the recording began. A trial consisted of enacting the entire sequence of the ten jokes one time through. Participants always began with one practice trial and then continued to enact two trials of ten jokes each in sequence. After these three trials, participants took a break and the recording was paused. During the break, they were reminded to say their lines to their partner with intent and not rush. Participants were then instructed to change their facing direction for another three trials including one practice trial.

After recording the joke telling trials, the participants completed a set of questionnaires on separate computers. Information about participants’ level of acquaintance with their partners and their experience of the experiment was gathered first. Participants were asked to rate how cooperative they found the other participant and how much they enjoyed telling jokes on a 9-point scale. In order to measure the participant’s social skill, they then completed the Autism Spectrum Quotient (Baron-Cohen et al., 2001) followed by the Self-Monitoring Scale (Snyder, 1974). The Autism Spectrum Quotient was developed to evaluate the degree to which an adult with normal intelligence exhibits traits associated with autism [see Baron-Cohen et al. (2001)]. The questions assess five areas of core autistic symptoms, *social skills*, *communication skills, imagination*, *attentional switching* (strong focus), and *attention to details*. The Self-Monitoring Scale evaluates how well and how motivated people are at regulating public expressiveness to fit the requirements of a social situation (Snyder, 1974). A high self-monitor is someone who behaves in ways that reflect the social norms of the situation, and is sensitive to others’ reactions both emotionally and behaviorally.

### DATA PROCESSING

#### Evaluating activity in the video recordings

A custom Matlab (Mathworks, Inc., Natick, MA, USA) script was used to calculate the amount of pixel change between adjacent video frames [see Schmidt et al. (2012)]. The amount of pixel change between adjacent video frames corresponds to the amount of bodily activity of a participant if they are the only source of movement in that part of the frame [see also Kupper et al. (2010), Paxton and Dale (2013)]. The Kinect camera collected the video images at an average frame rate of 22 Hz. In order to recover evenly spaced time-steps in the collected pixel change data, the cubic-spline interpolation function was applied to the data to resample them at 15 Hz. Compared to other methods, spline interpolation produces the best function shape in frequency domains as well as the least blurring effect in image recovery (Hou and Andrews, 1978; Meijering, 2000). The result was two activity time series, one for the joke teller and one for the joke responder, sampled at 15 Hz for each trial.

#### Cross-spectral coherence and relative phase analyses

To assess the degree and pattern of synchronization across all time scales, weighted coherence and the relative phasing of the two activity time series were evaluated (Schmidt and O’Brien, 1997; Richardson et al., 2005). Using a cross-spectral analysis, which allows one to determine the degree of correlation between two time series at each component frequency, the bidirectional weighted coherence (Porges et al., 1980; Goldfield et al., 1999) was calculated across the frequency band from 0.11 to 2 Hz which captures time scales of the rhythms inherent in the activity time series. The weighted coherence is a weighted average measure of the correlation (actually an *r*^2^ value) of the two time series across this frequency range and ranges on a scale from 0 to 1. A coherence of 1 reflects perfect correlation of the movements (absolute synchrony) and 0 reflects no correlation (no synchrony).

To assess the pattern of the coordination across all time scales, the instantaneous relative phase was calculated using a Matlab routine that calculated the relative phase angle for each sample of the time series. This was done by calculating the difference between the instantaneous phase angles of each participant’s movement using a Hilbert transform [see Pikovsky et al. (2001) for details about this transformation]. The calculated relative phase time series were then analyzed for the degree of attraction to the equilibrium points of a coupled oscillator model (i.e., 0 and 180^∘^) by finding the frequency of occurrence of the relative phase angles in each of nine 20^∘^ relative phase regions between 0 and 180^∘^. The resultant distributions of relative phase could then be used to evaluate whether dynamical phase entrainment occurred by determining whether there were concentrations of relative phase angles near the coupled oscillator system’s equilibrium points of 0 or 180^∘^ (i.e., inphase and antiphase modes).

#### Cross-wavelet analysis.

As pointed out by Schmidt et al. (2012), a spectral decomposition of the activity time series in such a joke telling task indicates that certain subsidiary rhythms at nested time scales including the time to recite one joke and the time to say one line (utterance) of the joke. To assess the degree and pattern of the participants’ movement coordination at individual time scales, a cross-wavelet analysis was used. A wavelet analysis is a time-frequency based analysis that allows one to perform a spectral decomposition continuously across time so that how the spectrum changes for each point in time can be estimated (Grinsted et al., 2004; Issartel et al., 2006). It is useful for complex time series with non-stationarities such as the activity time series of the participants’ movements. Wavelet plots (see, **Figure 4A**) display the amount of power (indicated by colors where blue is low power and red is high power) at each time scale (*y*-axis) for each point in time (*x*-axis) of the trial. A cross-wavelet analysis evaluates the cross-spectrum of two time series across time, and hence, can uncover how the time-localized coherence and relative phase at different frequency ranges (time scales) changes across the course of a trial. A cross-wavelet coherence plot (see **Figure 4B**) displays the coherence (correlation between the speaker and listener indicated by colors where blue is low correlation and red is high correlation) at each time scale (*y*-axis) for each point in time (*x*-axis) of the trial. In our analyses, we used a Morlet wavelet of order 8 to evaluate the coherence and relative phase at the time scales associated with the four characteristic events of the joke telling task: the joke, the half joke, the two-person exchange and the utterance. Because there were 10 jokes told in each trial and each joke seems to be comprised of eight utterance beats, the periods of these time scales were calculated as: joke period = trial time/10, half joke = joke period/2, 2-person exchange = joke period/4 and utterance period = joke period/8. After the cross-wavelet transform was performed, the mean of the coherence and the circular mean of the relative phase at these subsidiary time scales were extracted.

#### Creating virtual pairs

Finally, to evaluate whether the degree and pattern of synchronization across and within time scales of the activity time series was different from the degree and pattern expected by chance synchronization, “virtual pair” time series were created to form control conditions. These were obtained by combining the time series of one person in a pair with the times series of the other participants who they did not interact with but who stood in the spatial location of their partner while in the same motion and direction conditions. When the time series were of unequal lengths, the longer time series was truncated to equal the length of the shorter time series because the analysis required time series of equal length. The analysis of these virtual pair time series provided estimates of chance coordination that may occur between individuals when they were not affecting one another’s movements by presence and engagement but just saying the same jokes in sequence.

#### Statistical considerations

For the statistical analyses, Greenhouse–Geisser adjustments for violations of sphericity were made as necessary. In the post-analyses, simple effect *F*-tests were used to analyze interactions and a Bonferonni criterion was used to determine significant differences between individual means.

## RESULTS

The average length of a trial, which was comprised of the recitation of the ten knock–knock jokes, was 59.94 s (SD = 7.14 s). Because there were 10 jokes, this indicates that participant pairs spent on average 6 s in the telling of each jokes.

### ACTIVITY ANALYSIS

In order to evaluate whether the amount each participant moved in the telling of the jokes varied with the conditions, a measure of average pixel change for a trial was submitted to a three-way ANOVA with within-subjects variables of participant Role (teller, responder) and facing Direction (toward, away) and a between-subjects variable of Motion (gesture, non-gesture). The analysis revealed that the participants moved more when they told the joke [teller: 530, responder: 473; *F*(1,14) = 5.95, *p* = 0.03, η_p_^2^ = 0.30], when they were instructed to gesture [gesture: 560, non-gesture: 443; *F*(1,14) = 6.42, *p* = 0.03, η_p_^2^ = 0.31], and when they were facing each other [toward: 524, away: 479; *F*(1,14) = 7.8, *p* = 0.02, η_p_^2^ = 0.36]. However, the main effect of Direction was qualified by an interaction between Direction and Motion [*F*(1,14) = 6.79, *p* = 0.02, η_p_^2^ = 0.33]: The greater movement for facing toward was true when the subjects gestured (*p* < 0.002) but not when they were not told to gesture (*p* > 0.05). In summary, the joke teller moved more than the responder and both participants moved more when they gestured particularly when they faced toward each other. These results are perhaps not all that surprising although they bolster the validity of the pixel change video flow measure to capture the bodily activity that occurred in the interaction.

### CROSS-SPECTRAL COHERENCE ANALYSIS

To evaluate whether the bodily movements of the joke teller and responder were coordinated, the overall cross-spectral coherence of the two activity time series was submitted to a three-way ANOVA with a within-subjects variable of facing Direction (toward, away) and between-subjects variables of Condition (experimental pairs, virtual pairs) and Motion (gesture, non-gesture). Importantly, the analysis yielded a significant main effect of Condition [*F*(1,28) = 73.93, *p* < 0.0001, η_p_^2^ = 0.73] in which the coherence of the experimental pairs (*M* = 0.35) was found to be significantly greater than the chance coherence (*M* = 0.09). Additionally, a significant interaction between Direction and Condition [*F*(1,28) = 4.26, *p* < 0.05, η_p_^2^ = 0.13] demonstrated that the coordination in the experimental pairs was greater when they were facing toward one another than facing away (*M* = 0.40 vs. *M* = 0.31, *p* = 0.01) but that this was not true of the virtual pairs (*M* = 0.09 vs. *M* = 0.10, *p*> 0.05). The interaction between Motion and Condition, although non-significant [*F*(1,28) = 1.05, *p* > 0.05, η_p_^2^ = 0.04], suggested that the coordination in the experimental pairs was greater when they were not gesturing (*M* = 0.38) compared to gesturing (*M* = 0.32) but that this was not true of the virtual pairs (*M* = 0.09 and *M* = 0.10, respectively).

### RELATIVE PHASE ANALYSIS

**FIGURE 2:**
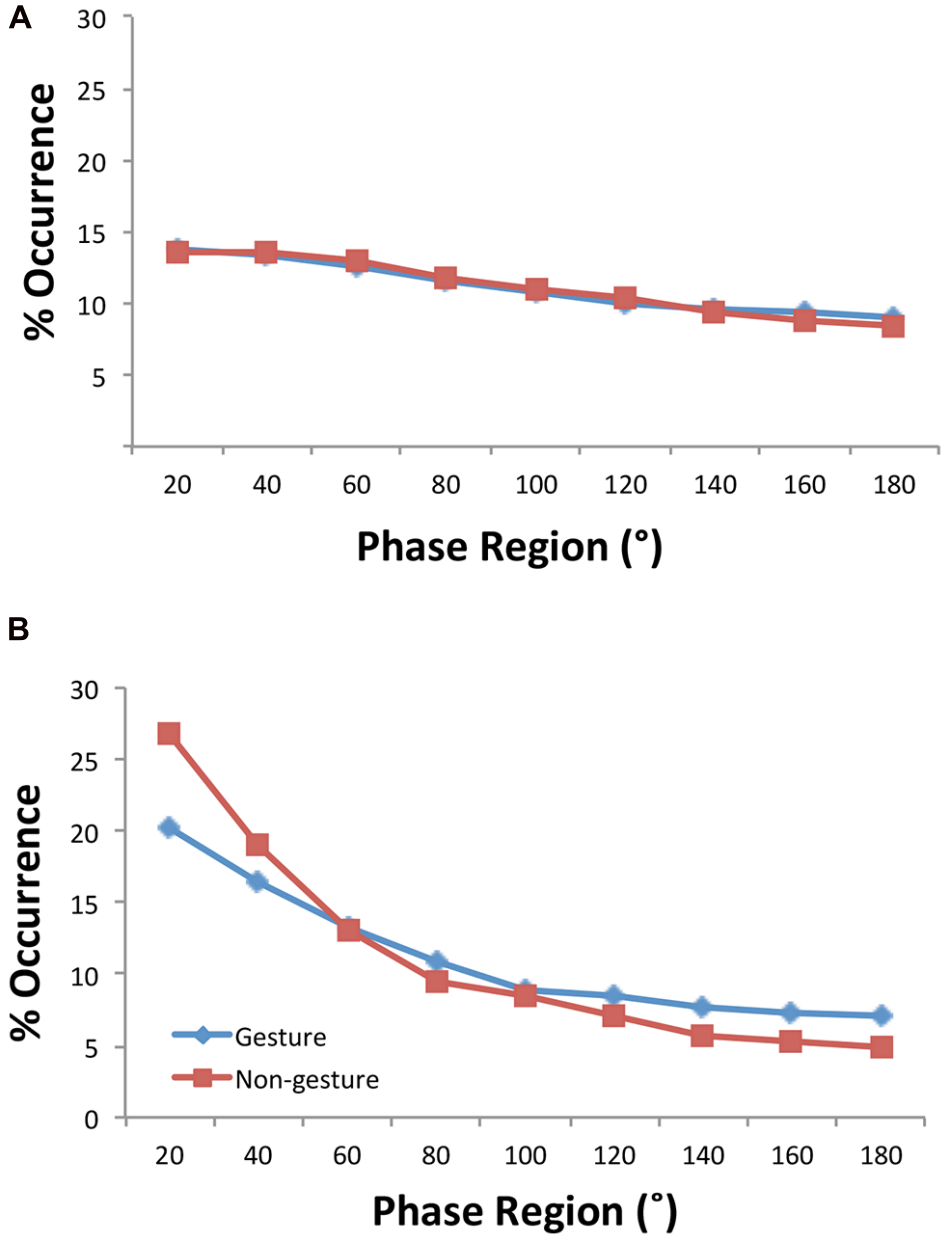
**Distributions of relative phase by motion conditions for the virtual **(A)** and experimental **(B)** participant pairs**.

**FIGURE 3:**
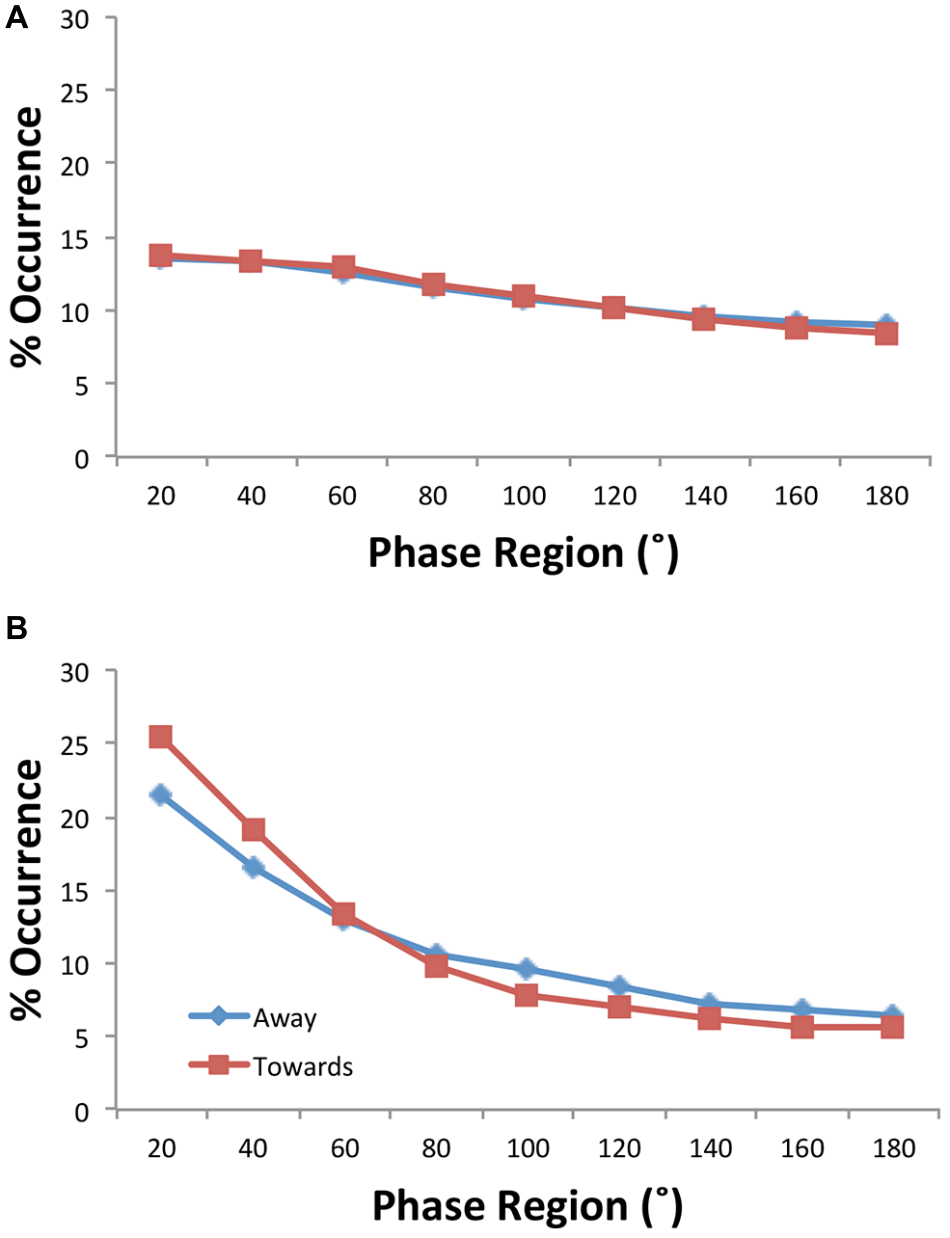
**Distributions of relative phase by facing conditions for the virtual **(A)** and experimental **(B)** participant pairs**.

Schmidt et al. (2012) discovered that in spite of the turn taking behavior inherent in joke telling that the relative phasing of activity tended to be inphase—that if one person moved the other person moved as well. To verify this inphase synchronization of movement and to see how it varied with the conditions manipulated, the distributions of relative phase were submitted to a four-way ANOVA with within-subjects variables consisting of facing Direction (toward, away) and the nine possible relative phase Regions (0–20, 21–40…, 161–180) and between-subjects variables of Condition (experimental pairs, virtual pairs) and Motion (gesture, non-gesture). Two three-way interactions summarize the important effects. First, a significant Condition × Motion × Region interaction [**Figure 2**; *F*(1.53,42.79) = 3.41, *p* = 0.05, η_p_^2^ = 0.11] demonstrated that the experimental pairs, but not the virtual pairs, exhibited inphase activity and also exhibited greater inphase activity in the 0–20^∘^ and 20–40^∘^ regions for the non-gesture condition [0^∘^: *F*(1,28) = 8.52, *p* = 0.007); 20^∘^: (*F*(1,28) = 5.26, *p* < 0.03)]—that is, the condition in which the subjects moved less. Additionally, a Condition × Direction × Region interaction [**Figure 3**; *F*(1.53,42.8) = 2.4, *p* = 0.12, η_p_^2^ = 0.08] indicated that the experimental pairs, but not the virtual pairs, exhibited inphase activity and exhibited greater inphase activity when the participants were facing toward each other (at 0 and 20^∘^: *p*s < 0.05).

### CROSS-WAVELET ANALYSES

These coherence and relative phase analyses evaluated the coordination that emerged between the joke teller and responder at all time scales. As pointed out by Schmidt et al. (2012), a spectral decomposition of such time series indicates that these behavioral waves seem to contain subsidiary rhythms at nested time scales including the time scales of the joke (i.e., every ∼6 s for the current data) and the utterance (∼6 s/8 utterance beats = every 0.75 s). Using a cross-wavelet analysis, we can evaluate the correlation between the two activity time series (i.e., their coherence) as well as their relative phasing at the different frequency bands associated with the time scales of these subsidiary rhythms. In addition to these macro and micro event time scales, we can evaluate the possibility of coordination as well as at intermediate event time scales, namely, the times scales that are related to the half joke (set up vs. punch line = ∼6 s/2 = every 3 s) and that of the two-person exchange (∼6 s/4 = every 1.5 s). A cross-wavelet analysis was performed on the activity time series of the joke teller and responder and the average coherence and relative phase angle were calculated at the time scales associated with these four nested events of the joke.

The wavelet coherence was submitted to a four-way ANOVA with within-subjects variables of facing Direction (toward, away) and Time Scale (joke, half joke, 2-person exchange and utterance) and between-subjects variables of Condition (experimental pairs, virtual pairs) and Motion (gesture, non-gesture). In addition to a significant main effect of Condition [*F*(1,28) = 109.58, *p* < 0.0001, η_p_^2^ = 0.80], the analysis revealed a significant Direction × Condition interaction [*F*(1,28) = 6.46, *p* = 0.02, η_p_^2^ = 0.19] and a non-significant Motion × Condition interaction [*F*(1,28) = 1.71, *p* > 0.05, η_p_^2^ = 0.06], which together verify the overall coherence results reported above. The analysis also revealed a significant main effect of Time Scale [*F*(1,28) = 12.75, *p* < 0.0001, η_p_^2^ = 0.31] that indicates that events at the longer time scales were more correlated (*M* = 0.42) than at the shorter time scales (*M* = 0.37, 0.36, and 0.37, all *p* < 0.01). However, this effect held true for both the experimental and virtual pairs (Condition × Time Scale: *F*(1,28) = 1.63, *p* > 0.05, η_p_^2^ = 0.06) which seems to suggest that greater correlations at the longer times is something that is related to how the participants were moving generally and not specifically how they were coordinated.

Note though that the analysis of wavelet coherence also provided some evidence of a Condition × Motion × Time Scale interaction [*F*(2.07,57.9) = 2.26, *p* = 0.11, η_p_^2^ = 0.08]. A simple effects analysis of the virtual pair data (**Figure 5A**) provided no evidence for differences between gesturing and non-gesturing at any time scale (all *p* > 0.05 and all η_p_^2^ < 0.05); however, a simple effects analysis of the experimental pair data (**Figure 5B**) demonstrated greater coherence for non-gesturing at the utterance [*F*(1,28) = 4.97, *p* = 0.03, η_p_^2^ = 0.15] and 2-person exchange time scales [*F*(1,28) = 2.79, *p* = 0.11, η_p_^2^ = 0.09] but greater coherence for gesturing at the joke time scale [*F*(1,28) = 2.62, *p* = 0.12, η_p_^2^ = 0.09]. These results seem to suggest that the non-significant all time scale coherence Motion and Condition interaction reported above (with non-gesture coherence being greater than gesture coherence) may have been non-significant because the effect only occurs at the faster event time scales of the utterance and two-person exchange and that gesturing may create greater correlations between the activity rhythms at the longer time scale of the joke (every 6 s).

The wavelet relative phase was submitted to a four-way ANOVA with within-subjects variables consisting of facing Direction (toward, away) and Time Scale (joke, half joke, two-person exchange and utterance) and between-subjects variables of Condition (experimental pairs, virtual pairs) and Motion (gesture, non-gesture). This analysis revealed a main effect of Condition [*F*(1,28) = 6.65, *p* = 0.02, η_p_^2^ = 0.19] as well as a Condition × Time Scale interaction [*F*(2.45,68.6) = 3.21, *p* = 0.04, η_p_^2^ = 0.10]. The latter effect (**Figure 6**) showed for the virtual pairs similar values near 0^∘^ relative phase for all four time scales [*F*(3,28) = 0.20, *p* > 0.05, η_p_^2^ = 0.02]; however, for the experimental pairs, the analysis demonstrated significant leading of the joke teller’s movements at the longer time scales [joke: *F*(1,28) = 11.02, *p* = 0.003, η_p_^2^ = 0.28], half joke: [*F*(1,28) = 3.05, *p* = 0.09, η_p_^2^ = 0.10]. Seeing this kind of leading at these time scales is what one would expect give that the joke teller initiates each joke at the period of the joke time scale and initiates the punch line at the period of the half joke.

### ANALYSIS OF SOCIAL COMPETENCE MEASURES

To examine whether the degree of bodily coordination exhibited by the participant pairs was related to the participant’s social competence, the overall coherence as well as the wavelet coherence at different time scales were correlated with the participants’ scores on the Self-Monitoring Scale as well as the Autism Spectrum Quotient and its subscales. Although neither the teller’s nor the responder’s Self-Monitoring scores were significantly correlated with any of the coherence measures, the responder’s scores on the Autism Spectrum Quotient were significantly correlated with the overall coherence (*r* = -0.60, *p* < 0.02) and the coherence of the two-person exchange event (i.e., 1.5 s time scale; *r* = -0.65, *p* < 0.01). These results suggest that as more autistic characteristics were present in the responder, the dyad exhibited a lower degree of entrainment. To further evaluate this effect, each the subscales of the Autism Spectrum Quotient were individually assessed for their relationship with bodily coordination. The social skills and communication skills subscales were not significantly correlated with any of the coherence measures. However, the attention to detail subscale was correlated with the coherence of the two-person exchange events (*r* = -0.61, *p* = 0.01) and the coherence of the utterance events (*r* = -0.61, *p* = 0.01) for the joke teller. Furthermore, the attention switching subscale was correlated with the overall coherence (*r* = -0.53, *p* < 0.05) and the coherence of the two-person exchange events (*r* = -0.53, *p* < 0.05) for the joke responder.

## DISCUSSION

Following Schmidt et al. (2012), we used bodily activity ascertained using pixel change measure to investigate the behavioral entrainment that arises from the social “dance” of a structured conversation joke telling task. Using virtual pairs as a control group, the results of the current study verify with both overall coherence and relative phase measures that indeed greater than chance behavioral entrainment occurred between the bodily movements of the teller and the responder of the jokes. Moreover, the magnitude of the coherence (*M* = 0.35) and relative phase occurrence of near inphase (*M* = 23.5%) are in the same range as those seen in Schmidt et al. (2012) as well as those in previous studies investigating spontaneous interpersonal entrainment using more stereotyped moving tasks such as swinging pendulums (Schmidt and O’Brien, 1997) and rocking in rocking chairs (Richardson et al., 2007). These magnitudes suggest that a weak dynamical coupling (Schmidt and Richardson, 2008) in which a relative coordination of rhythms (von Holst, 1973; Kelso and Ding, 1994) is apparent, and consequently, verify that bodily movements in conversation interactions can exhibit dynamically based interactional synchrony (Condon and Ogston, 1967; Newtson, 1993).

**FIGURE 4:**
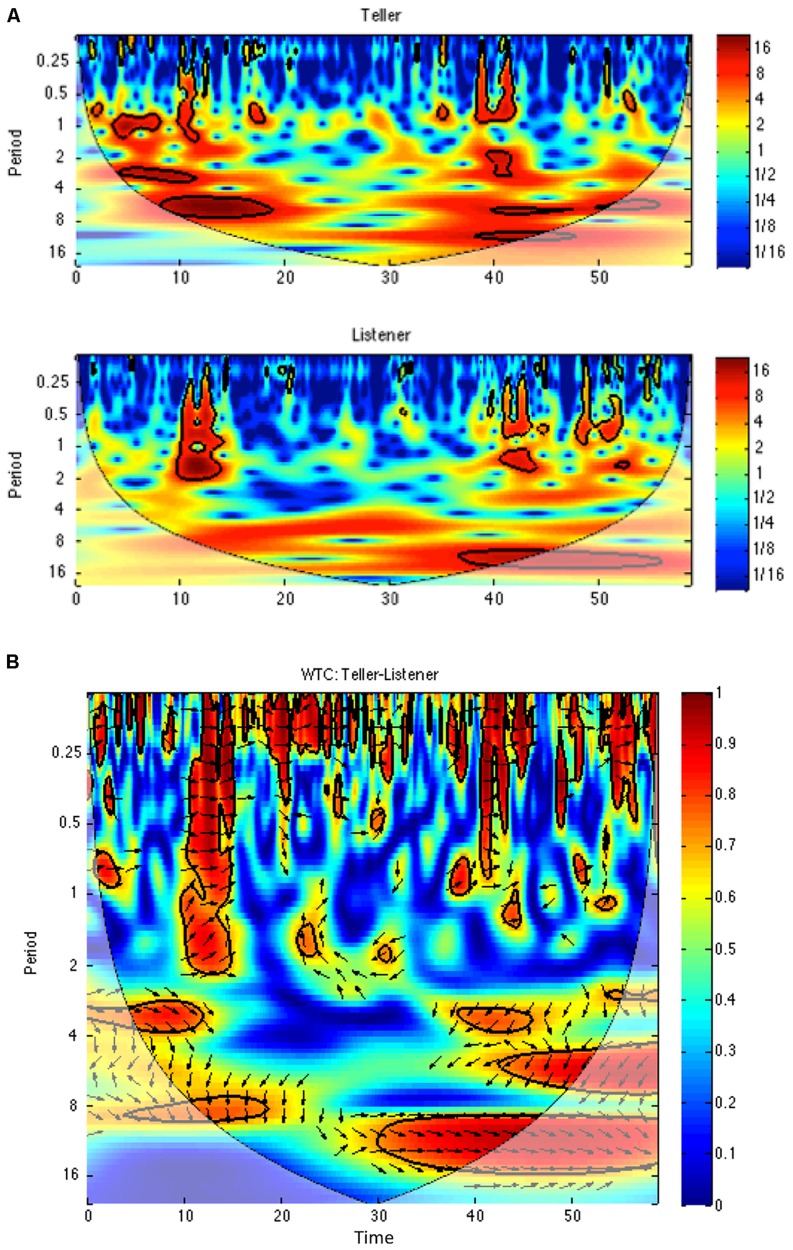
**Wavelet power **(A)** and cross-wavelet coherence **(B)** for an exemplary trial.** The length of the trial was 60 s (*x*-axis); consequently, the joke time, half-joke, two-person exchange and utterance time scales were 6, 3, 1.5, and 0.75 s (*y*-axis periods). In the top plot, note power has concentrations at these joke event time scales. In the bottom plot, coherence magnitude and relative phase at a given time scale and a point in time are denoted by color and the orientation of the arrow (pointing right: teller–listener inphase; left: teller–listener antiphase; down: teller leading by 90^∘^), respectively. The average of these values at a given time scale were extracted from these plots to perform the time scale coherence and relative phase analyses.

**FIGURE 5:**
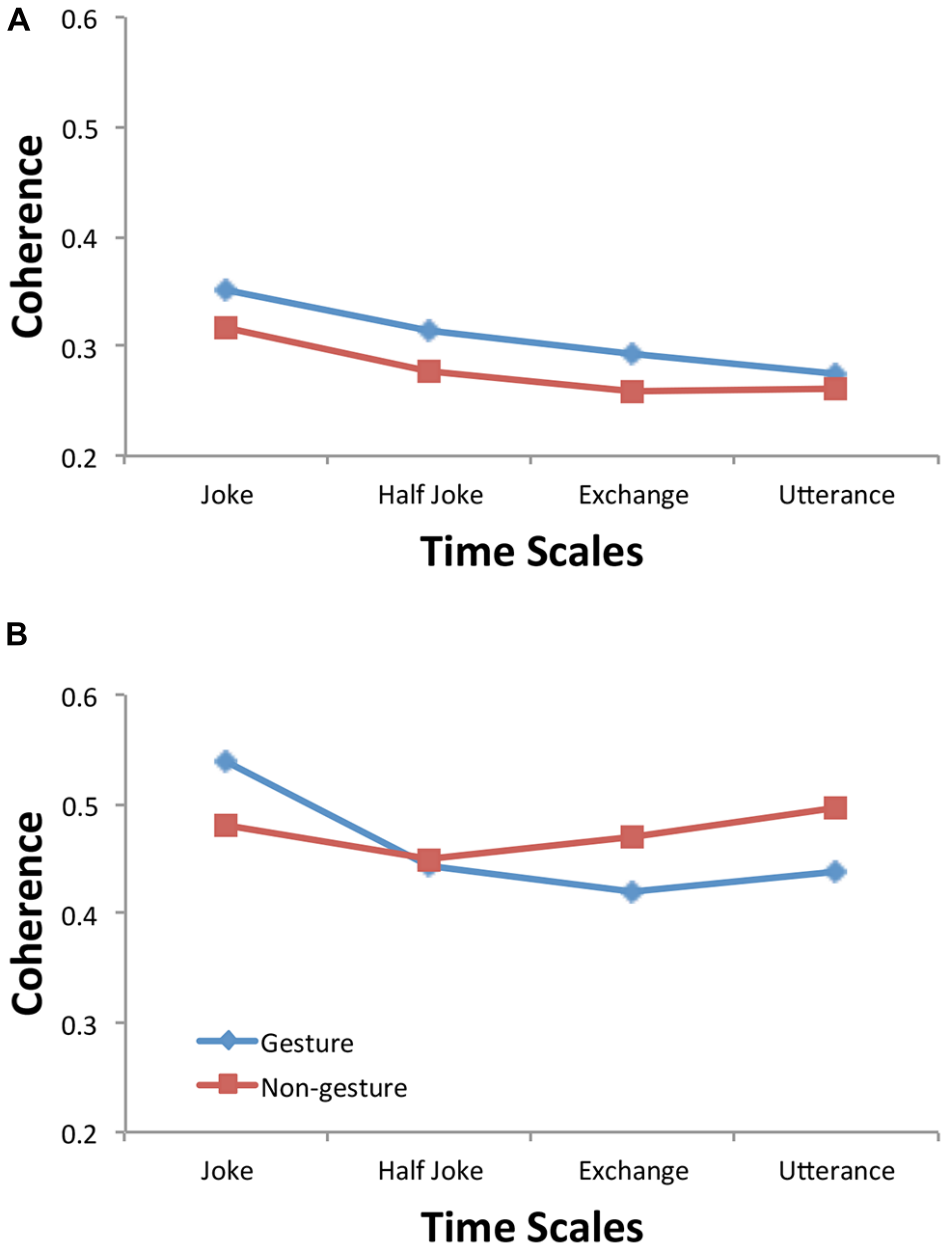
**Wavelet coherence at the four joke time scales by motion conditions for the virtual **(A)** and experimental **(B)** participant pairs**.

Moreover, the results also demonstrate for the first time that this behavioral entrainment of bodily movements occurs at different time scales. Underlying this observation is the view that the temporal nature of behavior needs to be defined in terms of hierarchically nested events/subgoals that comprise it (Schmidt, 2007): time is not just past, present and future but that the true chronons—units of time—of all nature are the inherent events that comprise a physical process (Fraser, 1978). The physical process or “dance” associated with knock–knock joke telling has a nested temporal structure in which the utterances, the two-person exchanges, and the set up and punch line were periodic events/sub-goals nested within the jokes themselves. The question was whether entrainment of bodily rhythms would occur for these subsidiary rhythms—whether indeed the temporal interaction between co-actors occurred not so much moment-to-moment but in terms of the dynamics of the behavioral events that make up the interaction. The cross-wavelet analysis (**Figure 4**) allowed us to extract the coherence and relative phasing of the joke teller and responder’s behavioral waves at these time scales. As can be seen in **Figure 5** and was demonstrated by the main effect of Condition, the coherence values at all four time scales for the experimental pairs (0.51, 0.45, 0.45, and 0.47) were greater than for the virtual pairs (0.34, 0.30, 0.28, and 0.27). This demonstrates that the experimental participant pairs synchronized above and beyond the virtual pair baseline synchronization (which captures the inherent relatedness of the joke’s intrinsic rhythms) for all the subsidiary rhythms that correspond to the nested events making up the enacting of the jokes. These results not only support the idea suggested by Newtson (1993) that social interactions consist of “coupled behavioral waves” but that these coupled behavioral waves are richly choreographed at nested time scales—the interactional synergy of these occurs not just moment-to-moment but rather in the dynamics of both long- and short-term events.

**FIGURE 6:**
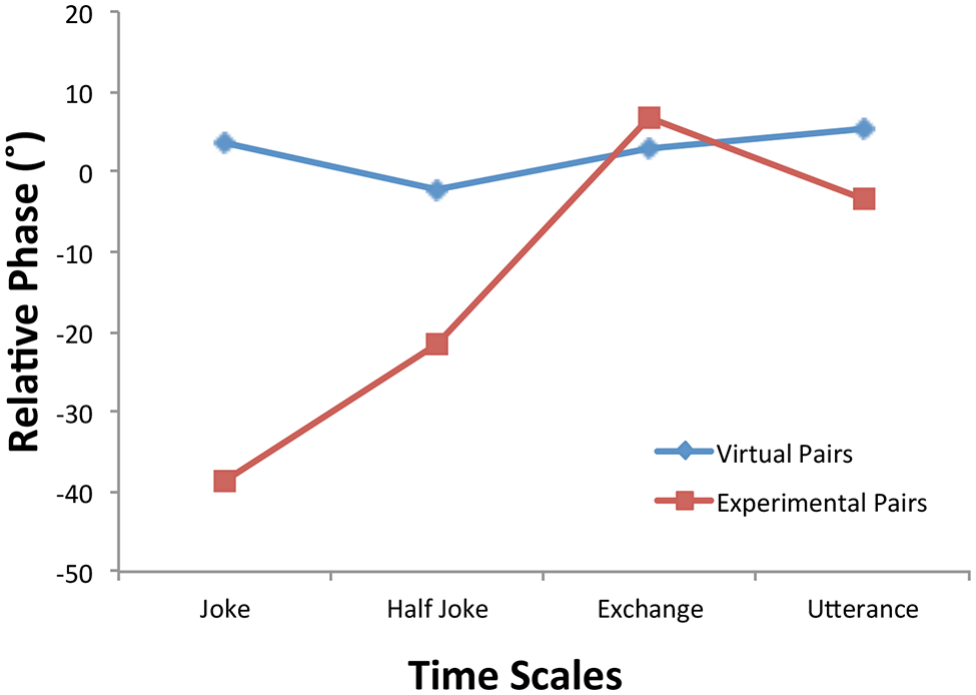
**Wavelet mean relative phase angle at the four joke time scales for the virtual and experimental participant pairs**.

An interesting aspect of the joke telling task is that the participants played different roles in the interaction: one person is the teller who initiates the joke and says the punch line while the other person is responder/appreciator of the joke. The mutual creation of a synchronous synergy of movement is constructed out of their performing complementary actions. This is different from previous investigations of interpersonal entrainment involving pendulums and rocking chairs in which the actions of the two people interacting were identical and the task imposed no systematic role differences. The breaking of this role symmetry had two observed effects in the current study. First, the results of activity ANOVA revealed that the joke teller moved more than the joke responder and did so whether they were told to gesture or not. Evidence of this result can be observed in the top panel plots of **Figure 4** in which the teller plot has greater power (deeper reds) throughout the trial than the listener. Second, the analysis of the wavelet relative phasing of the activity at the different time scales (**Figure 6**) demonstrated that for the two longer time scales the joke teller’s activity led that of the responder in time. The negative relative phase angles (joke: -39^∘^; half-joke: -21^∘^) indicate that for these joke events, the teller’s bodily activity led the responder’s by 10 and 5% of a cycle or 600 and 150 ms, respectively. Of course this leading in body movement of the speaker is perhaps a consequence of their leading in speaking in that previous research that has found a relationship between speaking and bodily motion: the act of speaking induces correlated postural sway and interpersonally coordinated speaking induces interpersonally coordinated body movements. (Shockley et al., 2007).

We also manipulated whether the participants told the jokes while facing toward or facing away from one another. Having visual information of each other not only increased the participants’ activity (especially when they were told to gesture) but also seemed to increase their degree of coordination as measured by the overall and wavelet measures of coherence as well as the distribution of relative phase angles observed (**Figure 3B**). Hence, participant pairs were more strongly entrained when they could see each other than when they could not, a result that continues to attest to visual information as a powerful medium to realize and sustain coordination (Schmidt et al., 1990; Richardson et al., 2007; Oullier et al., 2008). Perhaps just as important is the fact that when the participants were facing away, their bodily movements were still significantly entrained: visual information was not necessary for bodily coordination to occur. This finding replicates that of Shockley et al. (2003) who found that just verbal information (i.e., talking but not seeing) is sufficient for creating spontaneously coordinated postural sway of two conversing participants. In a follow-up study, Shockley et al. (2007) found that verbal coordination of posture does not occur on the basis of perceived speech signals but is dependent upon the rhythmic nature of the coordinated speech productions, which apparently induce coordinated postural movements. A similar mechanism is likely at work here given the coordination of speaking implicit in the joke structure (e.g., the turn taking) and the intimacy between speaking and body movement: the bodily coordination we observed during the facing away may not be due to the perceived speech signals but rather dependent upon structure of the joke task and the rhythmic nature of the coordinated speech productions.

Half of our participant pairs were instructed to explicitly gesture while telling (i.e., imitate knocking on a door) and responding (i.e., shrug shoulders and turn up the palms) to the joke. This manipulation was performed to increase the activity of the participants in telling the joke. Of interest was whether such an increase would naturally lead to greater entrainment in that the movements would be more visually perceivable. The activity analysis did reveal a main effect of Motion indicating that this manipulation did increase the activity for both the teller and the responder. However, interactions associated with the overall relative phase (**Figure 2B**) and coherence across the joke’s time scales (**Figure 5B**) indicate that explicitly increasing gesturing actually decreased the amount of entrainment observed, particularly, for the shorter time scales of the joke. While the relative phase analysis replicated for both gesturing and non-gesturing the inphase relationship previously shown to dominate participants’ activity during joke telling, the participant pairs moved more *together* (inphase) when enacting the jokes when they were not gesturing. The coherence across the joke’s time scales additionally specifies that gesturing seems to have obviated entrainment particularly during the faster, two-person exchange and the utterance events. It seems that in constraining the participants’ choices of gesturing by specifying the gestures to be used, we disturbed the inphase coordination of the participants movements that would naturally occur. This disturbance may have been due to having to attend more to their own gesture production, and hence, less to their partner’s gestures/movements. Alternatively, Bergmann et al. (2011) have shown that not all manual gestures are synchronized with the speech utterances they are to support: complementary gestures (e.g., drawing a circle when saying “clock”) compared to gestures that redundantly express the same information as the spoken words (e.g., drawing a circle when saying “round”) have been shown to have later onset time to their accompanying words. Although, it is unclear here whether imitating knocking when saying “Knock-knock” and shrugging when saying “Who’s there?” are complementary gestures, if the timing of the movements were not synchronized with their accompanying linguistic information, increased movement would not lead to greater interpersonal synchrony. Nonetheless, constraining the participants to produce specific gestures may have focused their attention toward the expressiveness/meaning of the speech and less toward their partner; hence, this would produce less coordinated activity.

A final goal of this study was to investigate whether the personality measures of social competence would relate to the degree of dynamical entrainment observed in the joke telling task. We measured the ability to self-monitor (Snyder, 1974), namely, the capacity to monitor one’s a social situation to ensure appropriate behavior, which has been argued to be a skill central to having successful social interactions and found to be related to nonverbal encoding and decoding skill (Riggio and Friedman, 1982). People high in self-monitoring are more likely to modify their behavior more based on the social situation, and consequently, are also more likely to take on leadership roles (Eby et al., 2003) in social groups. However, the results demonstrated that the degree of dynamical entrainment observed in our dyads was not associated with the degree of social monitoring of neither the tellers nor the responders. Of course, the lack of a relationship may be a consequence of the social monitoring measure evaluating social knowledge (i.e., what to do in a social situation) rather than the social process (i.e., the relative smoothness of social actions), which is perhaps what our measures of dynamical entrainment were indexing.

We also measured the social competence of our participants using the Autism Spectrum Quotient (Baron-Cohen et al., 2001), which has been used to investigate whether adults of average intelligence have autism-like symptoms. The measure has been used to verify that such symptoms exist in the normal population especially in people who have math and science skills. Although the Social Monitoring scale was unrelated to social motor entrainment in joke telling, we found moderate correlations between scores on the Autism Spectrum Quotient scale and both overall coherence and the two-person exchange event coherence. Furthermore, it was the more “process” related subscales of the Autism Spectrum Quotient scale, namely, attention to detail and attention switching, that were underlying these correlations rather than those, social skills and communication, that are arguably more associated with social knowledge. In essence, participants who had paid more attention to detail or had less ability to switch attention were found in the dyads that had a lower degree of social entrainment. This obviously points to the cognitive processes that constrain the formation and functioning of the interpersonal synergy that was formed and relates to previous research that has found peripheral or focal attentional attunement can affect the degree of interpersonal (Richardson et al., 2007) and environmental (Schmidt et al., 2007) entrainment. The Autism Spectrum Quotient results highlight that individual differences in this cognitive ability has consequences for social motor coordination in particular and the quality of social interactions and relationships in general in both normal and pathological populations (Fitzpatrick et al., 2013).

## CONCLUDING REMARKS

This study investigated the bodily “dance” underlying human communication interactions using a structured coordination task. Using behavioral dynamics methods, we found that the choreography of this everyday “dance” is complex: it contains coupled behavioral waves at nested temporal scales in which the complementary roles of the speaker and listener are apparent and which are supported in part by visual information and in part by verbal information. Moreover, we found that individuals vary in terms of their ability to create stable interpersonal entrainment and such skill seems to be underwritten by the flexibility by which they can attend to and pick up information about their environment.

Although the coupled activity waves studied here exist at the social/behavioral scale, behavioral dynamic investigations such as this provide a necessary constraint on the domain of two-person neuroscience (Hari et al., 2013). Because only when one can understand, model, and manipulate the system dynamics at the behavioral level can you begin to understand the roles that neural correlates, functional connectivity and neuroentrainment play in the creation of the bodily “dance” underlying human interactions. Indeed as stated in the introduction, behavior and mental states emerge from manifold interactions between the brain and environmental-situated behavioral consequences. Consequently, understanding the structure of the coordinated action of two people at the behavioral space-time scale is foundational for understanding the synchrony of their underlying neural processes.

## Conflict of Interest Statement

The authors declare that the research was conducted in the absence of any commercial or financial relationships that could be construed as a potential conflict of interest.
